# Targeting Abundant Fish Stocks while Avoiding Overfished Species: Video and Fishing Surveys to Inform Management after Long-Term Fishery Closures

**DOI:** 10.1371/journal.pone.0168645

**Published:** 2016-12-21

**Authors:** Richard M. Starr, Mary G. Gleason, Corina I. Marks, Donna Kline, Steve Rienecke, Christian Denney, Anne Tagini, John C. Field

**Affiliations:** 1 California Sea Grant Program, Moss Landing, California, United States of America; 2 Moss Landing Marine Laboratories, Moss Landing, California, United States of America; 3 The Nature Conservancy, Monterey, California, United States of America; 4 Fisheries Ecology Division, Southwest Fishery Science Center, National Marine Fisheries Service, National Oceanic and Atmospheric Administration, Santa Cruz, California, United States of America; Department of Agriculture and Water Resources, AUSTRALIA

## Abstract

Historically, it has been difficult to balance conservation goals and yield objectives when managing multispecies fisheries that include stocks with various vulnerabilities to fishing. As managers try to maximize yield in mixed-stock fisheries, exploitation rates can lead to less productive stocks becoming overfished. In the late 1990s, population declines of several U.S. West Coast groundfish species caused the U.S. Pacific Fishery Management Council to create coast-wide fishery closures, known as Rockfish Conservation Areas, to rebuild overfished species. The fishery closures and other management measures successfully reduced fishing mortality of these species, but constrained fishing opportunities on abundant stocks. Restrictive regulations also caused the unintended consequence of reducing fishery-dependent data available to assess population status of fished species. As stocks rebuild, managers are faced with the challenge of increasing fishing opportunities while minimizing fishing mortality on rebuilding species. We designed a camera system to evaluate fishes in coastal habitats and used experimental gear and fishing techniques paired with video surveys to determine if abundant species could be caught in rocky habitats with minimal catches of co-occurring rebuilding species. We fished a total of 58 days and completed 741 sets with vertical hook-and-line fishing gear. We also conducted 299 video surveys in the same locations where fishing occurred. Comparison of fishing and stereo-video surveys indicated that fishermen could fish with modified hook-and-line gear to catch abundant species while limiting bycatch of rebuilding species. As populations of overfished species continue to recover along the U.S. West Coast, it is important to improve data collection, and video and fishing surveys may be key to assessing species that occur in rocky habitats.

## Introduction

Management of multispecies fisheries that include stocks with various vulnerabilities to fishing has proven challenging for fisheries managers [1–5]. Prior to the start of more conservative fishery regulations in the U.S.A., management of many mixed-stock fisheries was often based on harvest rates and productivity estimates for abundant species that led to weaker, more vulnerable stocks being overfished [6–8]. Common life history characteristics of those more vulnerable species are traits such as slow growth, late age at maturity, and highly variable recruitment, which make them more susceptible to fishing pressure, particularly in multispecies fisheries or when non-selective fishing gear is used [9, 10]. In such scenarios, fisheries can remain economically viable as long as some target species remain productive, but this also can drive populations of less productive species towards collapse [4, 11, 12]. Failure to prevent the collapse of less productive stocks, coupled with the constraints necessary to rebuild those stocks, can result in considerable economic costs to the fishery, and have broad ecosystem impacts.

The U.S. West Coast groundfish fishery, comprised of more than 90 species of fish including flatfishes, rockfishes, roundfishes, and elasmobranchs, is an example of a fishery that experiences the management challenge of targeting abundant stocks while avoiding less productive stocks. Some commonly caught species are inherently vulnerable to overfishing, with long lifespans and late maturity, whereas others exhibit high productivity and are less vulnerable to high exploitation rates [3, 13, 14]. Attempts to maximize fishery yields in the 1970s and 1980s led to overfishing of many of the slower growing, less productive species [7]. Starting in the late 1990s, ten species of groundfish were declared overfished (as defined by an estimated spawning potential below 25% of the unfished level) by the U.S. Pacific Fishery Management Council.

Following U.S. federal fishery policies instituted in 1996 that required an end to overfishing, rebuilding plans have been put in place for overfished species. One such management measure was the implementation in 2002 of large depth-based, coast-wide fishing closures, known as Rockfish Conservation Areas (RCAs). The RCAs include an area closed to bottom trawling (the “trawl RCA”) along the entire U.S. West Coast that generally encompasses the area between the 183–274 m isobaths on the continental shelf-slope break and upper slope. Additionally, there is an area closed to commercial fixed gear (the “non-trawl RCA”) that approximately follows the 91–274 m isobaths on the shelf. Allowable depths for recreational fishing fluctuate annually, but are usually less than 60 m. In the last 15 years, the RCAs have been successful at reducing mortality of rebuilding species by protecting important habitats and reducing bycatch. However, three of the older-lived, less productive species [Cowcod (*Sebastes levis*), Pacific Ocean Perch (*S*. *alutus*), and Yelloweye Rockfish (*S*. *ruberrimus*)] will require considerably longer rebuilding timeframes (>20 years) and consequently will continue to constrain the fishery for abundant species, particularly as fishing opportunities open for previously overfished species [15].

In addition to closing fishing grounds inside the RCAs, causing fishing patterns to change [16], an individual fishing quota (IFQ) program was implemented in 2011 for the West Coast groundfish trawl sector that included hard caps on catch and 100% human observers for accountability. The limited quota available for rebuilding species provided a strong incentive to avoid these species, and bycatch and discards of rebuilding rockfish species dramatically decreased in the first few years of the IFQ program [17]. Although the RCAs and IFQ program have provided clear biological benefits [18, 19], those management measures decreased the amount of information available about the distribution, abundance, and size structure of overfished/rebuilding species with which to inform management and fishing decisions. This has resulted in substantial costs and limitations associated with the IFQ program, including very low quota levels for overfished/rebuilding species, that constrain the catch of other abundant stocks and limit access to fishing grounds due to the RCA area closures. These limitations have led to low catch levels of abundant stocks, with total catches ranging from 16–21% of the allowable catch for Lingcod, 21–36% of the allowable catch for Chilipepper (*S*. *goodei*), and 24–40% of the allowable catch for Yellowtail Rockfish (*S*. *flavidus*). As fishery participants are well aware, the result has been that both revenue and product supply have not yet recovered to their full potential.

The RCAs and other management measures have also had the unintended consequence of reducing the amount of fishery-dependent data available to assess population status of all fished species, an increasingly recognized challenge associated with the use of area closures to achieve management objectives [20–22]. With reductions in fishery-dependent data, the primary method of monitoring groundfish stocks is the annual West Coast Groundfish Bottom Trawl Survey [18, 23], which is conducted almost exclusively on low-relief, soft-bottom habitats. Unfortunately, the trawl survey often provides little information about most of the rebuilding rockfish species that inhabit high-relief, untrawlable habitats. Without directed sampling of all habitats used by rebuilding species, we continue to run the risk of misunderstanding the rebuilding trajectories and may unnecessarily constrain the harvest of robust stocks.

After 15 years of these large fishery closures, two major questions facing fishery managers are 1) how to determine the status of overfished stocks in areas that have been closed to fishing since 2002, and 2) how to improve utilization of abundant species while minimizing catches of overfished species. To address these questions, we designed and used a stereo-video camera system to collect fishery-independent information in untrawlable habitats, specifically areas that have not been surveyed since the implementation of the RCAs in 2002. We also conducted a collaborative study with fishermen in central California to test new fishing techniques to determine if abundant species can be caught without substantial bycatch of co-occurring rebuilding species. We paired the video and fishing surveys to examine populations of rebuilding species, relative to target species, in high-relief rocky areas in the same sites that we fished. We sampled areas and habitats not currently surveyed by the U.S. National Marine Fisheries Service.

## Methods

### Ethics Statement

The fishing portion of this research was conducted under an exempted fishing permit (#13-14-TNC-01) authorized by the U.S. Pacific Fishery Management Council. The Monterey Bay National Marine Sanctuary provided a permit for research activities involving the video surveys (MBNMS-2012-027).

### Site selection

**Fig 1:**
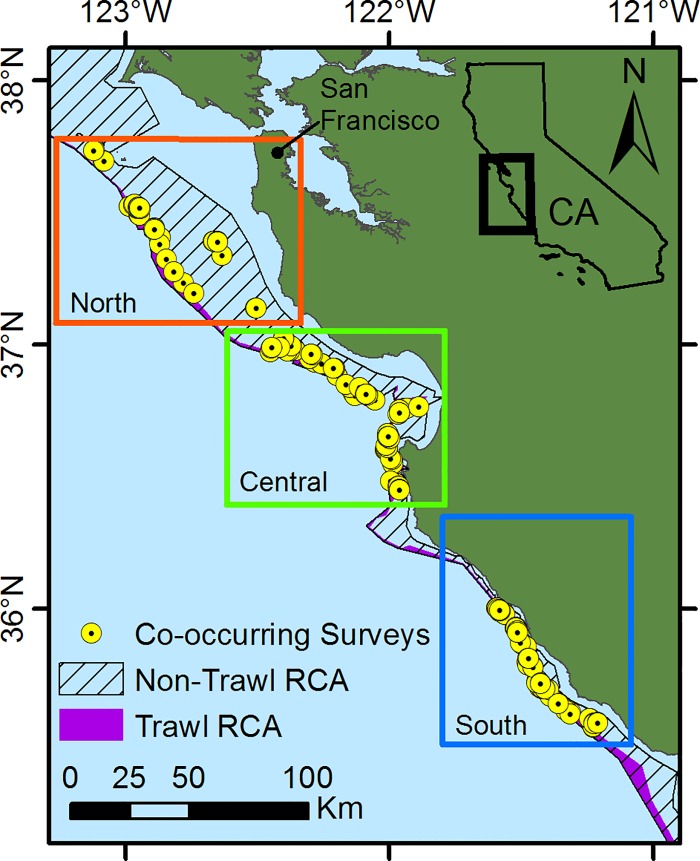
Map of locations of co-occurring video and fishing surveys in three sub-regions of central California, USA.

We conducted this study in central California (35.5° N to 37.8° N), U.S.A., between San Francisco and Morro Bay. We stratified the study area into three sub-regions (north, central, south) to account for regional variability and to distribute sampling effort throughout the study area. Within the three sub-regions we focused sampling effort on hard bottom habitats. We then conducted paired fishing and video surveys across and within those sub-regions (Fig 1), distributed across the depth ranges that generally correspond with the non-trawl RCA (55–183 m) and the trawl RCA (183–274 m).

### Video lander

**Fig 2:**
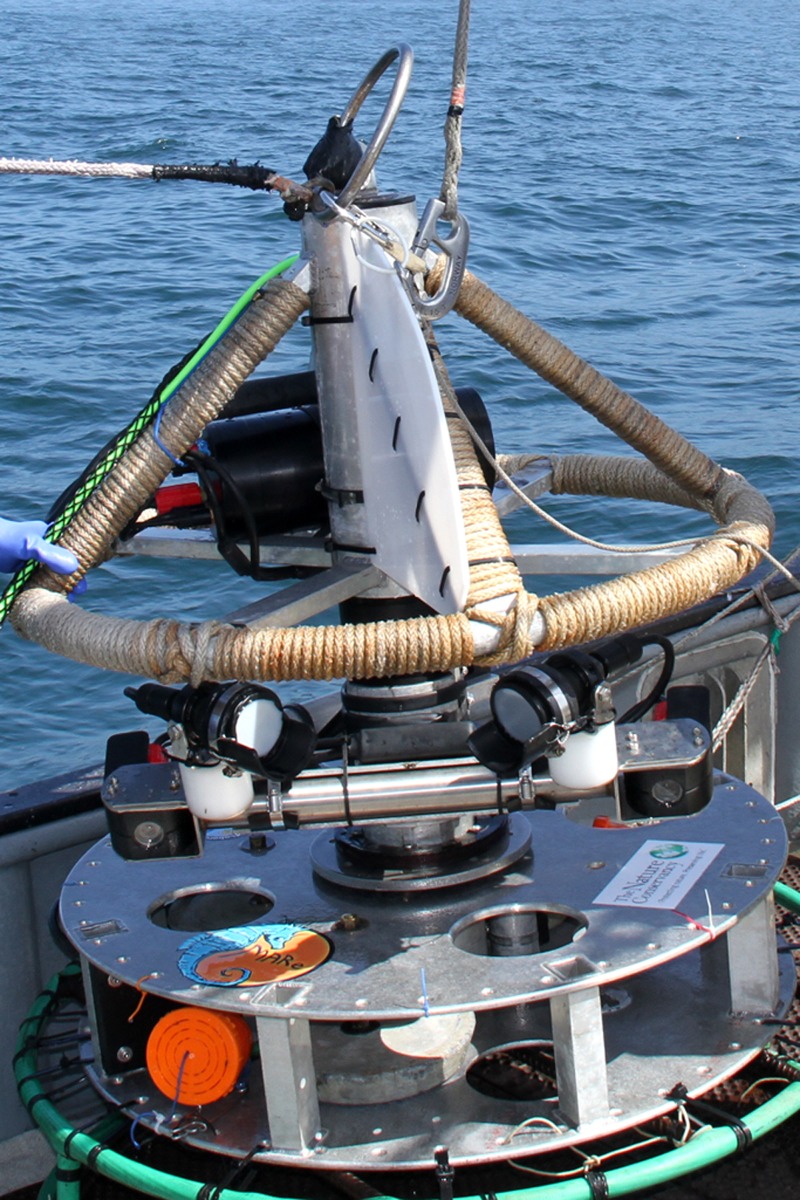
Stereo-video lander used to survey fishes in central California, U.S.A.

We developed and used an underwater camera system that consisted of paired video cameras mounted on a tethered lander capable of being deployed to depths of 300 m. We designed the tool to use “point count” visual protocols that were developed in the 1980s and continue to be used in quantitative assessment of fishery resources [24–26]. We used a pair of color, wide-angle, standard-definition video cameras (Deep Sea Power and Light Nano SeaCams) mounted obliquely on a rotating tray that enabled collection of stereo-video imagery of fishes and habitats in a 360-degree arc on and just above the seafloor around the stereo-video lander (Fig 2). The speed of the rotation was set so that each complete rotation took approximately one minute. The stereo-video lander was equipped with three dimmable LED lights, each providing 2600 lumens. A GoPRO camera (set at 1080p definition, medium field of view) was mounted above the main starboard camera during the second year to aid in species identification in the lab. Video files from the two stereo cameras were stored on hard drives at depth and also transmitted up an umbilical for real-time viewing and metadata collection onboard the support vessel. Prior to each cruise, we calibrated the stereo-video system in a test tank to ensure that length measurements were accurate.

### Video surveys

Video surveys were completed within one to two days of the fishing activities in each fishing site when weather permitted. On each sampling day we used a Simrad ES-60 echosounder to locate the area associated with the acoustic returns that the fishers targeted and to verify that the location we chose to sample contained rocky habitat. As our aim was to compare video surveys to baited fishing surveys, we attached two plastic bait jars containing chopped squid to the lander frame below the camera field of view.

In September and October of 2013 and 2014, we completed a total of 299 stereo-video lander surveys that were co-located with fishing surveys (Table 1). Of those 299 co-located video surveys, 124 occurred in the central sub-region, 110 in the south sub-region, and 65 in the northern sub-region. Surveys were conducted in water depths ranging from 70–250 m and the average depth of video surveys was 138.5 m. The majority of video surveys were conducted in depths shallower than 183 m due to difficulty locating rocky habitat in the deeper areas.

**Table 1:** Number of video surveys completed.

Year	North	Central	South	Total
2013	21	54	54	129
2014	44	70	56	170
Total	65	124	110	299

Number of video surveys completed within 500 m of experimental fishing sets by year and sub-region.

After deploying the video lander and allowing it to settle to the bottom, we waited 1–2 minutes for suspended sediment caused by the deployment to settle. We then recorded video of the fishes observed during the 360° rotation of the cameras for eight full rotations, then raised the lander off the bottom. Each of these occurrences was defined as one “survey”. Depending on the extent of rocky habitat and number of fishes observed, we conducted up to seven surveys per deployment. When we conducted multiple surveys in a single deployment, the lander was raised at least 10 m off the bottom, rotation and lights were turned off, and the boat transited at least 50 m from the previous survey location before lowering the lander to the bottom. Data collected from each survey included geographic coordinates, starting and ending times, fish counts, and species observed.

### Species composition, fish size, and density from video surveys

We used SeaGIS EventMeasure software (www.seagis.com.au) to obtain distances, lengths, and counts of fishes recorded in the video. Only surveys with eight complete 360° rotations of the cameras were used for analyses. We identified all fish to the lowest possible taxonomic level and recorded the maximum number of individuals (Max N) of each species present in the field of view for a single rotation during each survey [27, 28]. Similarly, we only measured the lengths of fish occurring in the rotation with the greatest number of observations for that species. Individuals that could not be identified to species level were grouped into higher taxonomic levels and counted in rotations where the highest number of that taxon was observed. All fish whose head and tail were clearly visible simultaneously in both cameras were measured using the EventMeasure software. After measuring fishes, we calculated the mean weight of each species using published length-to-weight relationships [13].

**Fig 3:**
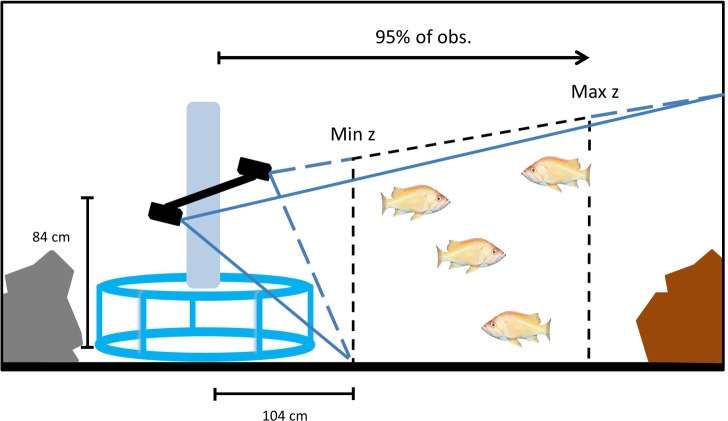
Schematic of area surveyed by rotating stereo-video cameras.

The stereo-video software enabled us to measure the distance from which fish targets were observed. For each species, we used a frequency distribution of measured distances to fishes to determine the distance within which 95% of all individuals were observed. These distances were deemed to be the maximum distances at which a video analyst could identify each species across a variety of seafloor conditions and only observations within that distance were used for density calculations. The minimum distance any fish could be observed (the closest point of the seafloor visible by the cameras) was 0.81 m away from the middle of the camera bar. We used this minimum distance and species-specific maximum distances from the cameras to calculate the area observed in each rotation (~25 m^2^), thus enabling us to calculate densities of each species in each survey (Fig 3).

### Experimental fishing

After discussions with fishermen, we decided to use vertical hook-and-line gear with multiple hooks that could be fished in a way to reduce interactions with rebuilding demersal species. Based on an earlier study in Oregon [29], we designed the fishing gear so there was a long leader, i.e., a space of 8 m between the bottom weight and the first hook. Also, we used fishing gear that provided instant notice of a fish biting a hook in order to avoid gear saturation. We used hydraulic “snapper reel” gear that allowed the baited hooks to be actively lowered and retrieved using a powered reel. The mainline was made up of 136 kg-test Dacron line. A 5–10 m length of 91 kg-test monofilament line that contained gangions and hooks was attached below the Dacron line by a swivel. Gangions were spaced approximately 30 cm apart along the monofilament portion of the mainline and each 15 cm-long gangion (45 kg-test line) contained a 10/0 hook baited with a strip of squid. An 8 m section of 82 kg-test nylon line, tied to a 4.5 kg weight, was placed below the monofilament line to serve as a breakaway section to minimize loss of hooks. The hook nearest to the bottom was kept more than 8 m above the 4.5 kg weight, and we attempted to keep the weight 2–3 m off the bottom to target fishing on species in the water column and to minimize contact of fishing gear with the bottom. We fished with one line at a time and each line carried 15 hooks. All fishing trips included a researcher who collected data on the catch, as well as a certified federal fishery observer, as required by conditions of the Exempted Fishing Permit.

At each fishing location, fishermen used their echosounders to search for target species (i.e., schooling fishes such as Chilipepper, Vermilion Rockfish, (*Sebastes miniatus*) and Yellowtail Rockfish) prior to setting gear. After deciding to fish, fishermen deployed the gear 3–5 times (sets) before moving to another location. Average set time equaled 5.1 ± 0.2 (SE) minutes and maximum set time equaled 20 minutes.

We conducted a total of 741 fishing sets over a 2-year period (Table 2), with 30 days and 416 completed fishing sets in 2013 and 28 days and 325 completed sets in 2014. The mean depth of fishing sets equaled 127 m, and depths of fishing sets ranged from 75–250 m. Fewer sets were made in depths ≥183 m because rocky habitat was not readily located at those depths and vessels were often unable to find suitable habitat on which to set the gear.

**Table 2:** Number of experimental fishing sets by year and sub-region.

Year	Northern	Central	Southern	Total
2013	79	112	225	416
2014	62	88	175	325
Total	141	200	400	741

At each fishing location, we recorded start and end depth, start and end geographic coordinates, time the gear reached the bottom, time the gear was retrieved, and bottom relief based on the vessel’s echosounder. As the gear was retrieved, we recorded the species caught on each hook to determine if there were patterns in catch by location along the vertical fishing line. We then measured each fish to the nearest 0.5 cm (total length) and weighed individuals or species groups. Fishermen were allowed to sell their catch (except rebuilding species) as an additional incentive to target desirable species as efficiently as possible during their fishing efforts.

We summarized the fishing data by year and sub-region to examine patterns in catch. We generated length frequency plots for key species from the two years of pooled data to determine what proportion of fish were at or above the size at 50% maturity [13, 23]. We then summarized the number of fishing sets that yielded rebuilding species and calculated the ratio of target to rebuilding species caught by year, using the cumulative weights for these species (target vs. rebuilding). We did not use the small volume (~1.1% by weight of the overall catch) of discards of non-target species for calculating target to rebuilding species ratios. Additionally, we compared the data from our fishing sets with information from commercial fishing landings in central California from 1990–1999. To evaluate the relative catch ratios of target–rebuilding species using comparable gear types, we extracted total commercial landings for shelf rockfish from all hook-and-line fisheries for the region between Morro Bay and San Francisco from the California Cooperative Commercial Survey database [30] for the period 1990–1999, which was just prior to the designation of overfished status for most of these species.

### Comparison of video and fishing surveys

**Fig 4:**
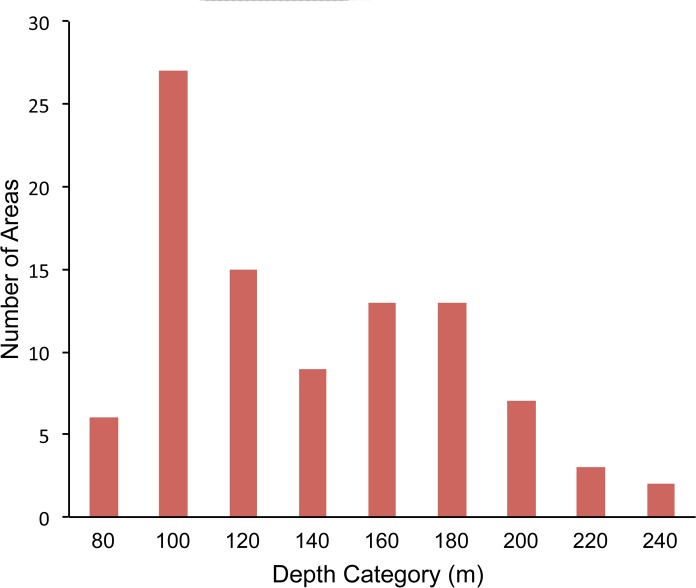
Distribution of the mean depths of the 96 areas containing paired video and fishing surveys.

We mapped fishing sets using the start and end coordinates of latitude and longitude recorded onboard during fishing operations. These coordinates were converted into vectors in ArcGIS and a 500 m buffer was drawn around each fishing set. Only video lander surveys within the 500 m buffer of a fishing set were designated as co-located with fishing. Fishing sets and video lander surveys were co-located in 96 discrete sampling areas, 57 of which we sampled in 2013 and 39 in 2014. The mean depths of the sample areas ranged from 72–237 m (Fig 4). On average, five fishing sets and three video-lander surveys occurred in each area. We defined these 96 discrete polygons of co-located fishing and video surveys as sample units for purposes of comparing video surveys and fishing sets. To determine if there was a relationship between what was caught and what was observed within a paired sample unit, we averaged both the number of fish caught per fishing set (CPUE) and the number of fish observed on video surveys for each of the paired samples and then transformed the data using a ln (x+1) transformation. We used correlation analysis to compare the transformed averages of fishing CPUEs and number of fishes observed per video lander survey.

In addition to correlation statistics, we used odds-ratio analysis [31] to answer the question of how the odds of catching a given species change if the species has been observed using the video lander. We considered a video observation of a species in a particular area as the treatment and catching that species in the same area as the positive “outcome”, and we tested the independence of treatments and outcomes. Also, we calculated a similar metric, the risk-ratio, to measure the change in probability of an outcome given a particular treatment, because risk-ratios are more intuitive to interpret than odds-ratio analysis [32].

Lengths of each fish measured, either collected by vertical hook-and-line fishing sets or stereo-video analysis, were pooled by species across the two years to produce length frequency distributions for selected species for each sampling technique. A Kolmogorov-Smirnov two-sample test was used to compare the length-frequency distributions for each species and sampling technique. Additionally, we calculated mean lengths from the video and fishing surveys for just those fish that were above the length at 50% maturity. We conducted a Welch’s t-test to compare mean lengths of fish in that size range. Also, we used a probability density function algorithm in the software package R to plot estimated frequency distributions of fishes from both fishing and video surveys.

### Comparisons of video, fishing, and trawl Surveys

The U.S. National Marine Fisheries Service conducted an average of 24 research trawl tows in our study area each year from 2003–2015, in the depth range of 70–250 m. We compared our fishing and video sampling results with data obtained from those annual bottom trawl surveys [23]. On May 16, 2016, we accessed the data portal (https://www.nwfsc.noaa.gov/data/map) for the annual trawl surveys that occurred in the region of our study, i.e., the central California coastal area from 35.5° N to 37.8° N latitude and in 70–250 m water depths. We downloaded information from the trawl surveys to enable us to compare research trawl data with our fishing and video surveys. We compared the weights and frequency of occurrence of fishes caught in trawl tows with weights and frequency of occurrence of fishes caught or calculated from lengths of fishes observed in our study.

## Results

### Video surveys

In the 299 video-lander surveys co-located with fishing sets, we observed a total of 10,873 fishes, representing 60 different species or species groups. A complete list of species observed in those 299 surveys, by sub-region, is included in **S1 Table**. Overall, 44% of video-lander surveys contained at least one rebuilding species. Lingcod was the species with the highest frequency of occurrence, and was recorded in more than 50% of surveys (Table 3). Vermilion Rockfish and Canary Rockfish (*S*. *pinniger*) each were observed in 27% of surveys, and Yelloweye Rockfish occurred in 21% of video-lander surveys. A total of 23 Cowcod were observed, representing 4% of video surveys. Both Yelloweye and Cowcod were observed more frequently in the south sub-region and less frequently in the north. Shortbelly Rockfish (*Sebastes jordani*), a small, semipelagic species of little to no commercial value was numerically the most abundant species observed, but they occurred in only 6% of video surveys. Many unidentified *Sebastes* spp. were observed at distances from the cameras that allowed general identification to family but not to species. Average density of all species combined in video surveys was approximately 1 fish/m^2^. Average density of rebuilding species was 0.13 ± 0.02 (SE) fish/m^2^.

**Table 3:** Total number of fish observed in video surveys for ten species of interest.

Common Name	Total	Freq. of occurrence
*Bocaccio	341	14%
*Canary Rockfish	678	27%
*Cowcod	23	4%
*Yelloweye Rockfish	98	21%
Chilipepper	110	6%
Lingcod	489	56%
Pacific Sanddab	371	12%
Vermilion Rockfish	1282	27%
Widow Rockfish	308	8%
Yellowtail Rockfish	541	23%

Total number of observations and the frequency of occurrence (Freq. of occurrence: percentage of surveys in which they occurred) for ten species of interest from the 299 co-located video surveys in 2013 and 2014. These species were chosen because they are commercially important, rebuilding species, or most abundant in video surveys, and were also caught in fishing sets.

* denotes rebuilding species.

### Experimental fishing sets

A total of 4,012 kg of fish was caught over the two fishing seasons (Table 4). Fish were caught on 294 of the 741 total fishing sets (39.7%); rebuilding species were caught in 80 of the 294 sets (27%) that caught fish. Although fewer fish were caught in the top third of the hooks, there were no significant differences among number of fish caught in the lowest, middle, or top third of the hooks deployed on the 15-hook fishing sets (ANOVA, df = 2, F = 2.45, p = 0.087). Overall, five out of 22 species caught comprised 98% of total landings by weight (Vermilion Rockfish, Yellowtail Rockfish, Chilipepper, Bocaccio (*S*. *paucispinis*), and Widow Rockfish (*S*. *entomelas*)). When we analyzed catches from just those species most commonly caught in shelf hook-and-line fisheries (i.e., Shelf Rockfishes, Table 5), the overall percentage of rebuilding species to all species caught in our project was 9.3%. Using those same species, the annual mean percentage of rebuilding species landed in central California commercial hook-and-line fisheries from 1990–1999 was 20.4% (Table 5).

**Table 4:** Weight (kg) of individual species caught and landed in fishing sets.

Common Name	Scientific name	2013	2014	Total
*Bocaccio	*Sebastes paucispinis*	191	126	317
*Canary Rockfish	*Sebastes pinniger*	9	15	24
*Cowcod	*Sebastes levis*	0	10	10
*Yelloweye Rockfish	*Sebastes rubberimus*	0	10	10
Bank Rockfish	*Sebastes rufus*	0	6	6
Blue Rockfish	*Sebastes mystinus*	0	26	26
Chilipepper	*Sebastes goodei*	113	320	433
Copper Rockfish	*Sebastes caurinus*	0	2	2
Greenspotted Rockfish	*Sebastes chloristictus*	2	23	25
Greenstriped Rockfish	*Sebastes elongatus*	0	0	1
Lingcod	*Ophiodon elongatus*	23	26	50
Pacific Sanddab	*Citharichthys sordidus*	10	28	38
Quillback Rockfish	*Sebastes maliger*	0	1	1
Sablefish	*Anoplopoma fimbria*	0	1	1
Sharpchin Rockfish	*Sebastes zacentrus*	1	0	1
Speckled Rockfish	*Sebastes ovalis*	4	11	15
Stripetail Rockfish	*Sebastes saxicola*	2	0	2
Vermilion Rockfish	*Sebastes miniatus*	1,335	1,076	2,410
Widow Rockfish	*Sebastes entomelas*	22	141	163
Yellowtail Rockfish	*Sebastes flavidus*	107	367	475
	Total	1,820	2,193	4,012

* denotes rebuilding species.

**Table 5:** Percentage of weight (kg per species) landed by commercial hook-and-line fisheries in central California from 1990–1999 [30] compared with percentages of species weights caught using experimental fishing gear 2013–2014.

Common Name	Mean Weight Commercial Shelf Rockfish Landings (kg) 1990–1999	Commercial % of Total 1990–1999	Total Weight (kg) Fish Caught this Project	This Project % of Total
*Bocaccio	639,905	11.0	317	8.1
*Canary Rockfish	344,012	5.9	24	0.6
*Cowcod	21,970	0.4	10	0.3
*Yelloweye Rockfish	181,256	3.1	10	0.3
Chilipepper	2,243,938	38.6	433	11.0
Greenspotted Rockfish	289,071	5.0	25	0.6
Vermilion Rockfish	617,641	10.6	2,410	61.2
Widow Rockfish	237,925	4.1	163	4.1
Yellowtail Rockfish	930,481	16.0	475	12.1
All Other	313,578	5.4	70	1.7
Total	5,189,977	100	3,937	100

* denotes overfished/rebuilding species.

### Comparisons of video and fishing surveys

**Fig 5:**
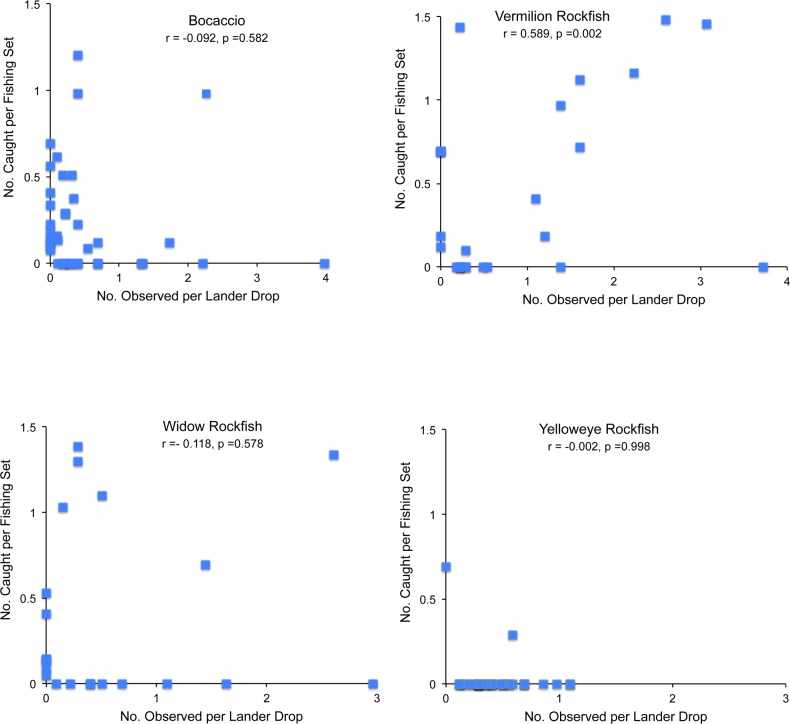
Example of the relationship between the number of fish caught in experimental fishing sets and the number observed in video surveys for Bocaccio, Vermilion Rockfish, Widow Rockfish, or Yelloweye Rockfish. Catches and observations were ln (x+1) transformed.

We caught rebuilding species in 46% of the 96 discrete areas of co-located fishing and video surveys. Most of the rebuilding species encountered in fishing sets, however, were Bocaccio and Canary Rockfish, which tend to be more mobile than other rockfishes. Yelloweye Rockfish were observed in 37 areas (39% of polygons) but caught in only two of those areas. Although two Cowcod were caught during fishing operations, no Cowcod were caught in areas in which fishing and lander surveys co-occurred. There was no significant relationship (p>0.05) between the number of rebuilding species observed near fishing sets and the number of rebuilding species caught during fishing operations (Table 6, Fig 5). There were, however, significant correlations between the number of target species caught (e.g., Vermilion Rockfish, and Yellowtail Rockfish) and the number of target species observed in co-located video surveys (Table 6).

**Table 6:** Correlation between the number of fish caught in experimental fishing sets and the number of fish observed in video surveys for target and rebuilding species. N represents the number of sample units in which a species was caught or observed.

Species	Correlation Coefficient	P-value	N
*Bocaccio	-0.092	0.582	38
*Canary Rockfish	0.012	0.951	27
*Cowcod	n/a		
*Yelloweye Rockfish	-0.220	0.184	38
Chilipepper	-0.239	0.167	35
Lingcod	0.038	0.771	61
Vermilion Rockfish	0.589	0.002	34
Widow Rockfish	-0.118	0.578	25
Yellowtail Rockfish	0.488	0.012	26

n/a signifies that no Cowcod were caught in fishing surveys located near video surveys. N represents the number of co-located fishing and video surveys (out of 96) in which a species was either caught or observed.

* denotes rebuilding species.

Odds-ratio (**S2 Table)** and risk-ratio (Table 7) analyses indicated that four species were more likely to be caught with commercial gear in areas where they were observed on camera. Yellowtail Rockfish exhibited the greatest increase in probability (risk-ratio = 62.48) of being captured when this species was observed on video in the same area. Yellowtail Rockfish was caught almost exclusively in areas where it was also observed on video surveys and was not caught in fishing sets in areas in which it was absent from the video surveys (Table 7). Canary Rockfish (risk-ratio = 24.23), Vermilion Rockfish (risk-ratio = 7.29), and Bocaccio (risk-ratio = 3.64) were all more likely to be caught if observed on video in an area. Conversely, Chilipepper, Cowcod, Lingcod, Widow Rockfish, and Yelloweye Rockfish did not have odds or risk-ratios significantly different than 1, indicating that the probability of catching these species with fishing gear did not change when these species were recorded from video surveys in the same area.

**Table 7:** Risk Ratios of catching a particular species, given that the species was observed in the same area by video lander.

Species	Risk Ratio	Lower 95% Confidence	Upper 95%Confidence	Fisher's p-value
*Bocaccio	3.64	1.86	7.11	< 0.001
*Canary Rockfish	24.23	3.23	182.00	< 0.001
* Cowcod	1.46	0.09	22.67	1
* Yelloweye Rockfish	1.59	0.10	24.73	1
Chilipepper	1.12	0.45	2.76	0.753
Lingcod	2.18	0.64	7.42	0.235
Vermilion Rockfish	7.29	3.23	16.42	<0.001
Widow Rockfish	3.24	1.39	7.57	0.017
Yellowtail Rockfish	62.48	8.89	439.80	<0.001

* denotes rebuilding species.

More species were observed in the video surveys than in the fishing sets (48 species identified from video versus 22 caught). We compared length frequency distributions for species in which sample size equaled 50 or more fish. For Bocaccio, Yellowtail Rockfish, Widow Rockfish, Vermilion Rockfish, and Chilipepper, Kolmogorov-Smirnov tests indicated that length frequency distributions were significantly different (p<0.05) between video surveys and fishing sets, primarily because more small fish were observed in video surveys. We also compared mean lengths of fishes that were larger than the length at 50% maturity (i.e., adult fish). In four of the five species evaluated, Welch’s t-tests indicated that mean lengths of fishes caught during experimental fishing sets were significantly larger than fishes observed in video surveys (Table 8).

**Table 8:** Comparison of mean lengths of adult fishes in both video and fishing surveys.

	Fishing Sets			Video Surveys				
Common Name	n	Mean	SE	n	mean	SE	p-value	
Bocaccio	122	59.0	0.9	148	51.1	0.7	<0.05	
Chilipepper	536	38.2	0.3	33	34.7	1.3	<0.05	
Vermilion Rockfish	1061	49.9	0.1	826	47.3	0.2	<0.05	
Widow Rockfish	117	40.9	0.3	40	41.0	0.5	0.844	
Yellowtail Rockfish	444	39.3	0.2	203	37.8	0.4	<0.05	

**Fig 6:**
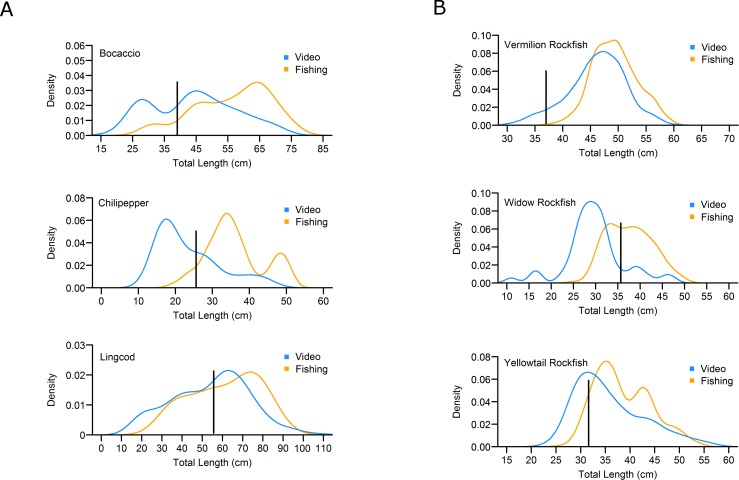
Length frequencies, expressed as kernel density distributions, of Bocaccio, Chilipepper, Lingcod, Widow Rockfish, Vermilion Rockfish, and Yellowtail Rockfish caught or observed in fishing or video surveys. The solid black line represents length at 50% maturity for females.

A broad size range was evident for Bocaccio, Chilipepper, and Lingcod (Fig 6A), and Widow Rockfish, Vermilion Rockfish, and Yellowtail Rockfish (Fig 6B). Except for Widow Rockfish and Chilipepper observed from video, the majority of fish caught or observed were either at or above the size of 50% female maturity [13, 23]. Nearly all Vermilion Rockfish observed or caught were larger than the size at 50% maturity. Similarly, most Yellowtail Rockfish and Bocaccio observed and caught were larger than the size at 50% maturity. Sub-adult Lingcod and Widow Rockfish were well represented in catches and often observed on video in relatively large groups of smaller individuals. Few mature Widow Rockfish were observed in video surveys. For all species that were both caught in fishing sets and observed in video surveys, we saw smaller individuals in the video surveys than were caught in the fishing sets.

### Comparisons of video, fishing, and trawl Surveys

**Fig 7:**
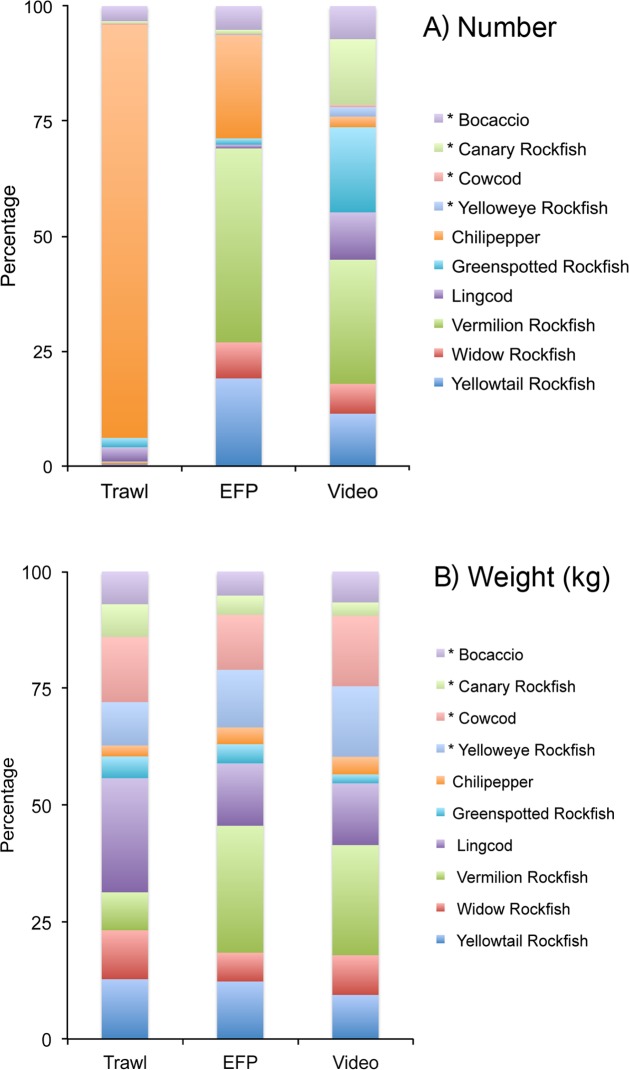
**Average species composition, by A) Number and B) Weight (kg) of fishes caught in National Marine Fisheries Service trawl surveys, and caught or observed in experimental fishing and video surveys conducted in this project.** Asterisks (*) denote rebuilding species.

When comparing the trawl data with our fishing and video surveys, the estimated average weights of fishes caught were similar among all survey techniques for Canary Rockfish, but were substantially different for the other primary species caught and observed in our fishing and video surveys (Table 9, Fig 7). Mean weights of Bocaccio caught in experimental fishing sets were more than twice that of fish caught in trawls or calculated from lengths of fishes recorded in video. Similarly, Cowcod were 3–7 times heavier in fishing and video surveys than in trawl surveys, and Widow rockfish were twice as heavy in trawl and fishing surveys as in video surveys.

**Table 9:** Estimates of the number and mean weight per fish of species caught or observed.

	Trawl		EFP		Video	
Species	N	kg/fish	N	kg/fish	N	kg/fish
* Bocaccio	2,150	1.1	130	2.4	341	1.0
* Canary Rockfish	332	0.9	22	1.2	678	0.9
* Cowcod	145	0.7	2	5.3	23	2.5
* Yelloweye Rockfish	12	2.1	4	2.6	98	1.4
Chilipepper	59,156	0.4	569	0.8	110	0.2
Greenspotted Rockfish	1,326	0.2	34	0.7	879	0.4
Lingcod	2,051	0.8	21	2.4	489	1.6
Vermilion Rockfish	187	1.2	1,066	2.3	1,282	1.6
Widow Rockfish	245	0.6	198	0.8	308	0.3
Yellowtail Rockfish	222	0.6	482	1	541	0.7

Estimates of the number (N) and mean weight (kg) per fish of species caught or observed per year in National Marine Fisheries Service trawl surveys conducted annually from 2003–2015 (Trawl), and from experimental fishing (EFP) and video surveys (Video) conducted in 2013 and 2014. Fishing and trawl data represent measured weights of fishes. Weights from video surveys are based on published length to weight relationships.

* denotes rebuilding species.

The number and percentage of samples in which species were present also varied by survey technique. Fishes occupying soft bottom habitats, such as Chilipepper and Lingcod, exhibited a higher frequency of occurrence in trawl surveys, which take place only in low-relief trawlable habitats, than in video surveys located in rocky habitats (Table 10). Conversely, for species that are more commonly associated with high-relief rock habitats, such as Canary Rockfish, Yelloweye Rockfish, and Yellowtail Rockfish, frequency of occurrence was higher in video surveys than trawl tows.

**Table 10:** Mean number of positive occurrences in trawl tows or video surveys.

	Trawl Surveys			Video Surveys		
	N Pos.		Avg. N	N Pos.		Avg. N
Species	Surveys	% FO	Fish/year	Surveys	% FO	Fish/year
*Bocaccio	7.8	33	165	41.9	14	171
*Canary Rockfish	2.5	11	26	80.7	27	339
*Cowcod	3.8	16	11	12.0	4	12
*Yelloweye Rockfish	0.5	2	1	62.8	21	49
Chilipepper	17.2	72	4,551	17.9	6	55
Greenspotted Rockfish	5.5	23	102	107.6	36	440
Lingcod	16.9	71	158	167.4	56	245
Vermilion Rockfish	1.6	7	14	80.7	27	641
Widow Rockfish	1.8	8	19	23.9	8	154
Yellowtail Rockfish	1.1	5	17	68.8	23	271

Mean number of positive occurrences in trawl tows or video surveys, the mean frequency of occurrence (% FO) in surveys, and the mean number of fish caught or observed per year for ten species of interest in National Marine Fisheries Service trawl surveys conducted annually from 2003–2015 (Trawl Surveys), and from video surveys conducted from 2013–2014 (Video Surveys). The mean number of trawl surveys each year equals 23.7 and the mean number of video surveys equals 149.5.

* denotes rebuilding species.

## Discussion

### Surveying Fishes in High-relief Habitats

The stereo-video lander system we developed proved to be a useful tool for assessing demersal fishes in moderate to high-relief rocky habitats. There were three primary outcomes of our work with respect to the potential utility of video lander data. First, there was a substantially greater frequency of occurrence of rebuilding species in video surveys, relative to trawl surveys. Second, our data indicated that estimates of fish abundance in low-relief habitats poorly reflected abundances of fishes that primarily occupy high-relief habitats. Third, the entire range of the size structure of fish populations is likely more appropriately sampled by *in situ* methods, because the video lander recorded a broader size range of individuals than did the trawls.

The primary tool used by the U.S. National Marine Fisheries Service to survey groundfish stocks is the West Coast Groundfish Bottom Trawl Survey [23]. This survey was initiated to provide fishery-independent data for stock assessments. The survey targets the commercial groundfish resources at depths of 55–1,280 m from Cape Flattery, Washington (lat. 48°10’N) to the U.S.–Mexican border (lat. 32°30’N). Approximately 750 trawl tows are sampled annually, offering an extensive fishery-independent dataset [23]. However, the trawl tows are conducted primarily in low-relief habitats, and provide only limited information about rebuilding species that live in high-relief habitats. This paucity of data collected from trawl surveys relative to species that are primarily associated with rocky or high-relief habitats is further exacerbated by the fact that areas with high abundances of rebuilding species are also closed to fishing, and fishery dependent data streams are therefore scarce. The inability to fully sample the appropriate habitat areas for many rockfish species contributes to uncertainty in estimates of both abundance and trend information, which are used in stock assessments [21, 22].

A key assumption made with respect to the bottom trawl survey data is that the catches of rebuilding species in low-relief habitats will be representative of the relative abundance of those species in areas that contain untrawlable habitats. Several researchers have suggested that this assumption may not be true if there are density-dependent processes driving the relative abundance of a given species across optimal (e.g., high-relief) versus suboptimal (e.g., low-relief or soft-bottom) habitats [33–35]. Our video surveys indicated that the abundance of Yelloweye Rockfish is probably much greater in central California than might be predicted based on research catches and/or bottom trawl surveys alone. Moreover, video surveys provided evidence of recruitment of Yelloweye Rockfish that was not apparent from trawl tows. Also, rebuilding species such as Yelloweye Rockfish were more abundant and had a broader size and age structure than was evident from trawl surveys alone. Not only was the frequency of occurrence of Yelloweye Rockfish ten times greater in video than trawl surveys, but also the trawl surveys captured an average of only one Yelloweye Rockfish per year in a >300 km stretch of coastline in central California. Indeed, due to such low catches, the trawl survey index used in the most recent Yelloweye Rockfish assessment was based on catch rates from Oregon waters alone, which were also low but considerably greater than those observed in central California [36].

Although the average number of Cowcod caught per year in trawl surveys was higher than the average number of Cowcod recorded in video surveys, the mean size of Cowcod recorded on video was three times higher than the size caught in trawls. Although stock assessment models will use selectivity curves to account for such differences, the models perform better when surveys can provide information on the entire size or age range of a population. The differences in mean sizes were likely due to species-habitat associations and ontogenetic movements. For example, Cowcod settle as juveniles into soft bottom habitats [37] and then disperse to deeper, typically high-relief rocky areas as adults [13]. Thus, an increase in numbers of Cowcod caught in trawl surveys may represent successful recruitment events but not be indicative of adult population sizes, and indeed visual surveys have proven to be more effective at developing accurate estimates of adult population size to inform assessment models for long-lived species such as Cowcod [38, 39].

The mean lengths of Bocaccio, Chilipepper, Vermilion Rockfish, and Yellowtail Rockfish caught were on average 2–8 cm larger than those observed in video surveys. We expected these results because fishing selects for the larger individuals in a population. When the two sources of data were combined, however, the length frequency distributions provided a more comprehensive estimate of the existing size classes of fishes in central California. Also, we note that many of the size frequency distributions contained a large proportion of both small fishes and fishes above the length at 50% maturity, which suggests the differences between fishing and video surveys are not likely to simply be a consequence of poor recruitment (resulting in larger mean lengths), given the observations of apparently high recruitment for most of these species in the 2008 through 2015 period [40, 41].

There has been a relatively long history of using manned submersibles to survey fishes on the U.S. West Coast [42], as well as remotely operated vehicles and stationary or towed cameras [43–47]. Unfortunately, video surveys have most frequently been local or regional in nature and few standardized surveys exist. Also, questions exist about the attraction and repulsion of fishes to light and sound emitted from submersibles, ROVs, and camera systems [48–50]. A clear need exists to standardize video surveys, as was done in the early days of developing standardized protocols for trawl surveys.

### Experimental gear performance

Populations of several demersal fishes have greatly increased along the U.S. West Coast since the implementation of the RCAs [19, 51, 52]. For example, the abundance of Lingcod in California waters was estimated to be at less than 10% of the unfished level in 1998, but above 70% by 2009 [53]. We worked with commercial fishermen to develop gear and techniques that can be more selective for the currently abundant species, such as Chilipepper, Vermilion Rockfish, Widow Rockfish, and Yellowtail Rockfish. Skippers attempted to fish the gear so that it remained vertical, to avoid fishes on the bottom. Our study yielded similar results as a previous study in Oregon that demonstrated that vertical fishing gear can catch semi-pelagic rockfishes while limiting catches of demersal rebuilding species [29]. In that study, catch rates of Yelloweye Rockfish and Canary Rockfish were reduced 84% and 41%, respectively, when fishing gear with a 3 m or 4.6 m long leader compared to gear with no long leader between the bottom weight and first hook. However, this analysis could not be replicated in our study due to the conditions of our experimental fishing permit.

Bocaccio was the one rebuilding species we did catch in moderate numbers, but the stock assessment for Bocaccio has shown an increase in biomass over the past decade, and the stock is currently projected to be above the target level in 2016 [54]. The correlation analyses we conducted between catches in an area and observations in co-occurring video surveys showed that when schooling Vermilion and Yellowtail Rockfish were abundant, our experimental fishing sets resulted in high catches. Also, we were clearly fishing on species in the water column, and not catching fishes off the bottom, as evidenced by the similarity in catch rates on hooks at different heights above the bottom. This is an important point, because we know that the species that were caught in the water column also occur on the bottom [13, 42, 46]; thus fish from rebuilding Cowcod and Yelloweye populations were not rising off the bottom to bite hooks.

An interesting part of the fishing/video comparison is that we found no significant relationship between catches and occurrences of Yelloweye Rockfish and Cowcod, the two rebuilding species that most limit the allowable catch of abundant species. This suggests that even in habitats where these demersal species are found in high numbers, higher abundance does not lead to higher catch rates using gear that is fished 8 m off the bottom. Therefore, as populations of those two species continue to recover, the fishing methods we used may provide a way to catch more productive and abundant species while limiting the bycatch of Cowcod and Yelloweye Rockfish. The success of the fishing technique in avoiding Cowcod and Yelloweye Rockfish is even more impressive, when considering that recent stock assessments indicate their populations are at higher levels now when they were declared overfished [36, 38].

## Conclusions

Our study demonstrated tangible benefits of combining experimental fishing activities with video surveys to more fully document the distribution, abundance, and size of rebuilding and selected target species within the RCAs. We were able to document that rebuilding species, including the ones such as Yelloweye Rockfish and Cowcod that most limit the allowable catch of abundant species, are distributed all along the central California region, but occur primarily in rocky or high-relief habitat. We also documented broad size ranges within their populations.

A comparison of fishing and video surveys indicated that fishermen could fish with modified hook-and-line gear to catch semi-pelagic species while rarely catching rebuilding species that were shown to be present in the video surveys. This suggests that as populations of overfished species start to recover in mixed-species demersal fisheries, there are ways to target abundant species while limiting bycatch of rebuilding species or less productive species.

## Supporting Information

S1 Table Video Survey Data. Complete list of fish species observed in video surveys that were co-located within 500 m of fishing sets (n = 299 lander surveys) by sub-region and depth category. Frequency of occurrence (Freq. occur.) is calculated from the number of surveys out of 299 in which the species was observed. Rebuilding species are denoted by an asterisk (*).
(DOCX)

S2 Table Odds Ratios. Odds ratios of catching a particular species, given that the species was observed on video in the same area. Lower and Upper 95% confidence intervals and Fisher’s Exact test results are reported.
(DOCX)
