# Supplementary material for: Targeting Abundant Fish Stocks while Avoiding Overfished Species: Video and Fishing Surveys to Inform Management after Long-Term Fishery Closures
Source: PLoS One. 2016 Dec 21;11(12):e0168645. doi: 10.1371/journal.pone.0168645 (PMC5176310; doi:10.1371/journal.pone.0168645)
Supplement: S1 Table — Complete list of fish species observed in video surveys that were co-located within 500 m of fishing sets (n = 299 lander surveys) by sub-region and depth category. Frequency of occurrence (Freq. occur.) is calculated from the number of surveys out of 299 in which the species was observed. Rebuilding species are denoted by an asterisk (*). (DOCX) [file pone.0168645.s001.docx]

**Table S1**. **Video Survey Data**

| **Genus** | **Species** | **Common Name** | **North** | **Cent.** | **South** | **Total** | **Freq. occur.** |
| --- | --- | --- | --- | --- | --- | --- | --- |
| *Sebastes* | *jordani* | Shortbelly Rockfish | 54 | 508 | 1066 | **1628** | 6% |
| *Sebastes* | spp. | Genus- Rockfishes | 408 | 490 | 630 | **1528** | 58% |
| *Sebastes* | *miniatus* | Vermilion Rockfish | 11 | 33 | 1238 | **1282** | 27% |
| *Sebastes* | *chlorostictus* | Greenspotted Rockfish | 517 | 306 | 56 | **879** | 36% |
| *Sebastes* | *pinniger* | Canary Rockfish* | 171 | 386 | 121 | **678** | 27% |
| *Sebastes* | *wilsoni* | Pygmy Rockfish | 10 | 262 | 275 | **547** | 27% |
| *Sebastes* | *flavidus* | Yellowtail Rockfish | 178 | 273 | 90 | **541** | 23% |
| *Ophiodon* | *elongatus* | Lingcod | 62 | 203 | 224 | **489** | 56% |
| *Citharichthys* | *sordidus* | Pacific Sanddab | 12 | 273 | 86 | **371** | 12% |
| *Sebastes* | *elongatus* | Greenstriped Rockfish | 268 | 77 | 10 | **355** | 26% |
| *Sebastes* | *paucispinis* | Bocaccio* | 185 | 95 | 61 | **341** | 14% |
| *Sebastes* | *entomelas* | Widow Rockfish | 143 | 160 | 5 | **308** | 8% |
| *Sebastes* | *sebastomus* | Subgenus - Sebastomus | 62 | 90 | 99 | **251** | 39% |
| *Sebastes* | *semicinctus* | Halfbanded Rockfish | 12 | 152 | 63 | **227** | 7% |
| *Eptatretus* | *stoutii* | Pacific Hagfish | 51 | 68 | 82 | **201** | 23% |
| *Sebastes* | *rosaceus* | Rosy Rockfish | 66 | 56 | 53 | **175** | 25% |
| *Sebastes* | *goodei* | Chilipepper | 56 | 51 | 3 | **110** | 6% |
| *Sebastes* | *constellatus* | Starry Rockfish | 34 | 44 | 27 | **105** | 20% |
| *Sebastes* | *ruberrimus* | Yelloweye Rockfish* | 21 | 30 | 47 | **98** | 21% |
| *Hydrolagus* | *colliei* | Spotted Ratfish | 4 | 12 | 72 | **88** | 10% |
| *Sebastes* | *caurinus* | Copper Rockfish | 27 | 44 | 9 | **80** | 13% |
| *Sebastes* | *hopkinsi* | Squarespot Rockfish | 8 | 27 | 39 | **74** | 11% |
| *Anoplopoma* | *fimbria* | Sablefish | 15 | 20 | 1 | **36** | 6% |
| *Sebastes* | *rubrivinctus* | Flag Rockfish | 7 | 21 | 6 | **34** | 7% |
| *Sebastes* | *saxicola* | Stripetail Rockfish | 17 | 3 | 6 | **26** | 3% |
| *Merluccius* | *productus* | North Pacific Hake | 11 | 5 | 9 | **25** | 4% |
| *Sebastes* | *levis* | Cowcod* | 1 | 7 | 15 | **23** | 4% |
| *Sebastes* | *mystinus* | Blue Rockfish | 19 | 0 | 0 | **19** | 1% |
| Pleuronectidae | spp. | Righteye Flounders | 4 | 9 | 3 | **16** | 4% |
| Bothidae | spp. | Lefteye Flounders | 0 | 0 | 11 | **11** | 1% |
| *Sebastes* | *melanostomus* | Blackgill Rockfish | 0 | 10 | 0 | **10** | <1% |
| *Hexagrammos* | *decagrammus* | Kelp Greenling | 5 | 4 | 0 | **9** | 2% |
| *Sebastes* | *rufus* | Bank Rockfish | 2 | 1 | 6 | **9** | 2% |
| *Zaniolepis* | spp. | Combfishes | 1 | 5 | 2 | **8** | 2% |
| *Argentina* | *sialis* | North-Pacific Argentine | 0 | 0 | 7 | **7** | <1% |
| *Microstomus* | *pacificus* | Dover Sole | 2 | 4 | 1 | **7** | 2% |
| *Sebastes* | *ensifer* | Swordspine Rockfish | 0 | 0 | 7 | **7** | 1% |
| *Sebastes* | *helvomaculatus* | Rosethorn Rockfish | 4 | 0 | 3 | **7** | 2% |
| *Parophrys* | *vetulus* | English Sole | 2 | 4 | 0 | **6** | 2% |
|  |  | Family – Smelts | 0 | 0 | 6 | **6** | 1% |
| *Sebastes* | *ovalis* | Speckled Rockfish | 0 | 3 | 2 | **5** | 1% |
|  |  | Family- Poachers | 0 | 3 | 2 | **5** | 1% |
|  |  | Family- Sculpins | 0 | 3 | 2 | **5** | 1% |
| *Eopsetta* | *jordani* | Petrale Sole | 2 | 2 | 0 | **4** | 1% |
| *Lycodes* | *cortezianus* | Bigfin Eelpout | 0 | 2 | 2 | **4** | 1% |
| *Rhinogobiops* | *nicholsii* | Blackeye Goby | 1 | 0 | 2 | **3** | 1% |
| *Sebastes* | *zacentrus* | Sharpchin Rockfish | 3 | 0 | 0 | **3** | <1% |
| *Anarrhichthys* | *ocellatus* | Wolf-eel | 0 | 1 | 1 | **2** | <1% |
| *Sebastolobus* | spp. | Genus- Thornyheads | 0 | 0 | 2 | **2** | <1% |
|  |  | Family- Pricklebacks | 2 | 0 | 0 | **2** | <1% |
| *Glyptocephalus* | *zachirus* | Rex Sole | 0 | 1 | 0 | **1** | <1% |
| *Lepidopsetta* | *bilineata* | Rock Sole | 0 | 1 | 0 | **1** | <1% |
| Bathymasteridae | spp. | Ronquils | 1 | 0 | 0 | **1** | <1% |
| *Porichthys* | *notatus* | Plainfin Midshipman | 0 | 1 | 0 | **1** | <1% |
| *Raja* | *rhina* | Longnose Skate | 0 | 0 | 1 | **1** | <1% |
| *Sebastes* | *aurora* | Aurora Rockfish | 0 | 0 | 1 | **1** | <1% |
| *Sebastes* | *crameri* | Darkblotched RF* | 0 | 0 | 1 | **1** | <1% |
| *Sebastes* | *maliger* | Quillback Rockfish | 1 | 0 | 0 | **1** | <1% |
| *Sebastes* | *nigrocinctus* | Tiger Rockfish | 1 | 0 | 0 | **1** | <1% |
|  |  | Unknown fishes | 41 | 121 | 45 | **207** | 26% |

Complete list of fish species observed in video surveys that were co-located within 500 m of fishing sets (n = 299 lander drops) by sub-region. Frequency of occurrence (Freq. occur.) is calculated from the number of drops out of 299 in which the species was observed. Rebuilding species are denoted by an asterisk (*).
