# Supplementary material for: Targeting Abundant Fish Stocks while Avoiding Overfished Species: Video and Fishing Surveys to Inform Management after Long-Term Fishery Closures
Source: PLoS One. 2016 Dec 21;11(12):e0168645. doi: 10.1371/journal.pone.0168645 (PMC5176310; doi:10.1371/journal.pone.0168645)
Supplement: S2 Table — Odds ratios of catching a particular species, given that the species was observed on video in the same area. Lower and Upper 95% confidence intervals and Fisher’s Exact test results are reported. (DOCX) [file pone.0168645.s002.docx]

**S2 Table. Odds Ratios of catching a particular species, given that the species was observed in the same area by video lander.**

| **Species** | **Odds Ratio** | **Lower 95% Confidence** | **Upper 95%**  **Confidence** | **Fisher's p-value** |
| --- | --- | --- | --- | --- |
| *Bocaccio | 6.69 | 2.46 | 18.21 | < 0.001 |
| *Canary Rockfish | 36.53 | 4.33 | 308.34 | < 0.001 |
| * Cowcod | 1.47 | 0.09 | 24.3 | 1 |
| * Yelloweye Rockfish | 1.61 | 0.10 | 26.57 | 1 |
| Chilipepper | 1.16 | 0.33 | 4.1 | 0.753 |
| Lingcod | 2.43 | 0.62 | 9.49 | 0.235 |
| Vermilion Rockfish | 18.6 | 5.95 | 58.16 | <0.001 |
| Widow Rockfish | 4.73 | 1.39 | 16.14 | 0.017 |
| Yellowtail Rockfish | 513.3 | 50.78 | 5189 | <0.001 |

Asterisks (*) denote rebuilding species.
